# Phosphorylation meets nuclear import: a review

**DOI:** 10.1186/1478-811X-8-32

**Published:** 2010-12-23

**Authors:** Jonathan D Nardozzi, Kaylen Lott, Gino Cingolani

**Affiliations:** 1 Dept. of Biochemistry and Molecular Biology, SUNY Upstate Medical University, 750 E. Adams Street, Syracuse, NY 13210, USA; 2 Dept. of Biochemistry and Molecular Biology, Thomas Jefferson University, 233 South 10th Street, Philadelphia, PA 19107, USA

## Abstract

Phosphorylation is the most common and pleiotropic modification in biology, which plays a vital role in regulating and finely tuning a multitude of biological pathways. Transport across the nuclear envelope is also an essential cellular function and is intimately linked to many degeneration processes that lead to disease. It is therefore not surprising that phosphorylation of cargos trafficking between the cytoplasm and nucleus is emerging as an important step to regulate nuclear availability, which directly affects gene expression, cell growth and proliferation. However, the literature on phosphorylation of nucleocytoplasmic trafficking cargos is often confusing. Phosphorylation, and its mirror process dephosphorylation, has been shown to have opposite and often contradictory effects on the ability of cargos to be transported across the nuclear envelope. Without a clear connection between attachment of a phosphate moiety and biological response, it is difficult to fully understand and predict how phosphorylation regulates nucleocytoplasmic trafficking. In this review, we will recapitulate clue findings in the field and provide some general rules on how reversible phosphorylation can affect the nuclear-cytoplasmic localization of substrates. This is only now beginning to emerge as a key regulatory step in biology.

## Introduction

### Principles of Nucleocytoplasmic Transport

**Figure 1:**
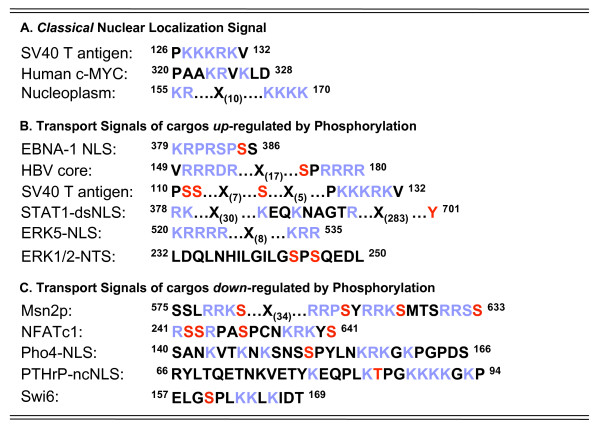
**Amino acid sequence of several known nuclear targeting sequences**. (**A**) Classical Nuclear Localization Signals. Nuclear import signals of cargos (**B**) up-regulated and (**C**) down-regulated by phosphorylation. Colored in blue are basic amino acids within the import signal, usually critical for recognition by karyopherins. In red are sites of phosphorylation that have been shown to modulate nuclear import.

The nucleus is the key organelle where most of the cellular genetic information is stored. Transcription is also compartmentalized to the nucleus to keep it separate from translation, which occurs in the cytoplasm. The nucleus is separated from the cytoplasm by a double lipid bilayer known as the nuclear envelope (NE), which is composed of an outer and an inner nuclear membrane (abbreviated as ONM and INM). At distinct points, the ONM and INM are conjoined by large macromolecular assemblies known as nuclear pore complexes (NPCs). Because of this peculiar morphology, all transport across the nuclear envelope is physically restricted to occur through the NPC. The NPC is not simply a channel, but mediates the exchange of macromolecules into and out of the nucleus imposing selectivity like a semi-permeable filter [1]. Accordingly, proteins smaller than ~40 kDa can usually diffuse through the NPC, while larger proteins are shuttled through the NPC by dedicated nuclear transport receptors of the β-karyopherin family, which includes at least 20 different known importins and exportins in humans [2]. Much is known about the molecular basis for the recognition of import/export cargos by β-karyopherins. As a general rule, substrates moving through the NPC typically expose molecular flags on their surface, thereby promoting efficient recognition and binding by karyopherins. Import cargos posses a Nuclear Localization Signal (NLS) that can be recognized by a karyopherin directly or in the presence of an adaptor protein (reviewed in [3-7]). Typically, NLSs are highly enriched in basic residues (e.g. Lysine, Arginine). Well-studied examples of classical NLSs are those of the SV40-Large T antigen [6], and the bipartite NLS of nucleoplasmin, which contains two basic clusters spaced by ~10 less conserved residues (Figure 1A). In contrast, cargos traveling toward the cytoplasm usually display a leucine-rich Nuclear Export Signal (NES), which contains critical hydrophobic residues, necessary for recognition by the nuclear export receptor Crm1[8].

β-karyopherins were originally identified based on their ability to bind the small GTPase Ran through an N-terminal binding domain [9]. Ran, a small GTPase of the Ras-superfamily, is recognized as the main source of both the energy and directionality of nucleocytoplasmic transport [10,11]. This GTPase exists in different nucleotide-bound states across the nuclear envelope. The nuclear population of Ran is predominately GTP-bound, whereas the cytoplasmic pool of Ran is GDP-bound due to the sequestering of its RanGEF (named RCC1) and RanGAP to the nucleus and cytoplasm, respectively [12,13]. The high concentration of Ran-GTP found in the nucleus is needed to dissociate incoming import complexes, and also to assemble outgoing export complexes.

**Figure 2:**
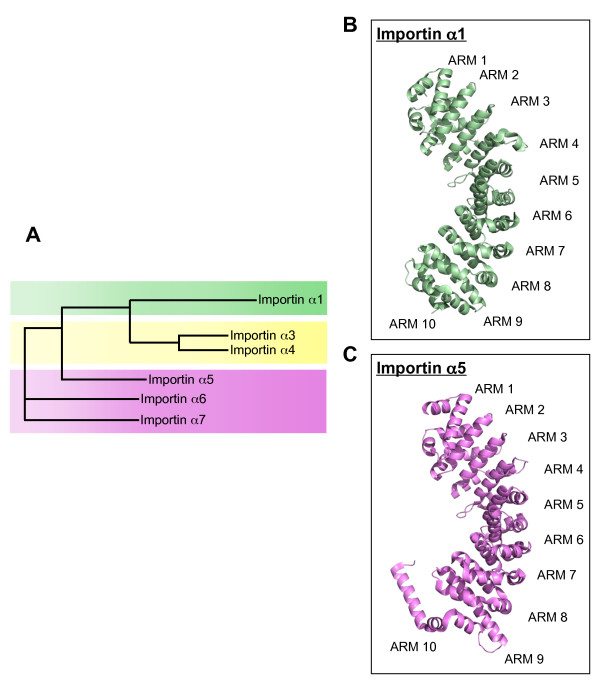
**Diversification of the human nuclear import adaptor importin α**. (**A**) A phylogenetic tree showing the evolutionary divergence of the six different human importin α isoforms. The branch lengths are proportional to the predicted evolutionary time between sequences. Three subfamilies of importin α (shaded in green, yellow and magenta) are identified. Both sequence alignment and phylogenetic tree were generated using the program ClustalW [164]. Ribbon diagram of the mammalian importin α1 (**B**) (pdb 1EJL) and importin α5 (**C**) (pdb 2JDQ) (in green and violet, respectively). Both structures consist of 10 tandemly repeated Armadillo repeats (ARM), each formed by three α-helices. Significant differences can be seen between importin α1 and α5 C-terminal ARM-10, which is partially extended in α5 [20]. Both crystal structures in panel (**B-C**) lack the N-terminal Importin β binding (IBB) domain, which promotes binding to the receptor importin β.

The most widely characterized β-karyopherin family member, importin β1 (reviewed in [2,14]) imports substrates directly or in complex with the adaptor proteins such as importin α or snurportin [15]. In the 'classical' nuclear import pathway [3-7], the adaptor importin α1 recognizes and binds a cytoplasmic cargo bearing a classical NLS. Importin α1 is tethered to importin β1 via its N-terminal importin β binding (IBB) domain [16] and the ternary complex of importin 1/importin 1/NLS is shuttled through the nuclear pore complex. Upon binding to RanGTP, the import complex falls apart, thereby releasing the import cargo in the cell nucleus, and importin β1 is shuttled back to the cytoplasm. In addition to importin α1, which recognizes classical NLSs, humans posses five additional isoforms of importin α, which fall into three distinct phylogenetic groups, namely the α1s, α2s and α3s [17,18] (Figure 2A). The functional diversification of importin αs occurred throughout evolution along with the evolution of multicellular animals. This reflects the increasingly complex and regulated nature of higher organisms and the need to perform cell and tissue specific functions during development and differentiation [17]. Whereas different importin α paralogs share the ability to bind and import *classical *NLS substrates only certain animal isoforms can recognize specific *non-classical *cargos. For instance, importin α5 (also known as NPI-1 is involved in the nuclear import of dimeric phosphorylated STAT1 [19] and influenza virus polymerase PB2 (Figure 2B) [20], while importin α3 (Qip-1) mediates translocation of NF-κB p50/p65 heterodimer into the nucleus [21].

In addition to the prototypical receptor importin β1, other β-karyopherins are well characterized. For instance, importin β2 (also known as transportin) mediates nuclear import of cargos containing an M9-signal [22]; or Crm1 recognizes NES-containing cargos in the presence of RanGTP and is responsible for their export out of the nucleus [3-7]. Overall, import and export complexes moving between the cytoplasm and nucleoplasm travel approximately 200 nm through the NPC to complete a round of translocation. This is a dynamic process that requires extensive interactions of β-karyopherins with the NPC.

### The Nuclear Pore Complex

It is impossible to appreciate the complexity of nucleocytoplasmic trafficking without acknowledging the morphology and composition of the NPC. In vertebrates, the NPC is a ~125 MDa macromolecular assembly composed of approximately thirty proteins, termed nucleoporins (nups) (reviewed in [23-26]). The nups are arranged into an octagonal symmetric pore that contains a central channel, sandwiched between a cytoplasmic and a nucleoplasmic ring of nucleoporins. These rings extend eight filaments deep into their respective cellular compartments. The cytoplasmic filaments, composed mainly of Nup358, extend flexibly into the cytoplasm and contain four RanBP1-like domains [27]. SUMO1 targets the Ran GTPase-activating protein RanGAP1 to Nup358, and together, these two proteins play a critical role in maintaining the Ran gradient across the nuclear envelope [13]. In contrast, the ends of the nucleoplasmic filaments, composed mainly of Nup153, are joined in a distal ring called the nuclear basket, which functions to arrest nuclear transport receptors awaiting cargo release in the nucleus [28]. About one third of nucleoporins in the NPC is highly enriched in phenylalanine-glycine repeats (FG-nups). These FG-nups are highly unstructured and are thought to be responsible for gating the NPC [29]. Increasing evidence indicates that the soluble FG-nups adopt a natively unfolded conformation [30] that may 'fill' the NPC inner channel and make it impermeable to cargos larger than ~40 kDa. Several models for translocation through the NPC have been proposed [30-33], although the lack of a direct way to study the movement of protein through the NPC hinders an accurate and quantitative characterization of the translocation mechanisms.

### Phosphorylation in Nuclear Transport

Reversible phosphorylation of amino acid side chains is the most widely characterized post-translational modification in biology (reviewed in [34-37]). Phosphorylation can either activate or inactivate biological pathways and is commonly used to switch enzyme activity "on" or "off". The enzymes responsible for addition and removal of a phosphate moiety, kinases and phosphatases, respectively, are highly abundant in nature. Genomics studies have shown that the human genome contains 518 protein kinases and only 147 protein phosphatase catalytic subunits, of which 107 belong to the Tyrosine phosphatase family [38]. This gives rise to countless networks of phosphorylation/dephosphorylation events that control the most diverse biological pathways. This review will focus only on the regulatory role of phosphorylation in nuclear transport.

Phosphorylation can have either an enhancing or an inhibitory effect on nuclear import, and unfortunately the molecular consequences of phosphorylation have been elucidated in very few systems [7]. Understanding how phosphorylation affects nuclear import is a difficult task and likely many more cargos than those few characterized to date (discussed in this review) are regulated by phosphorylation. It is generally accepted that cargos smaller than ~40 kDa can slowly diffuse into the nucleus, while larger cargos are actively imported by karyopherins. However, some of the smallest proteins in a cell (e.g. Hiv-1 Rev [39], PTHrP [40], histone H1 [41], etc.) use karyopherin-mediated, signal-dependent import pathways, clearly demonstrating that nuclear import is about the rate at which cargos enter the cell nucleus, and not their size. Unfortunately, the two most common assays used to study nuclear transport, the nuclear import assay in digitonin permeabilized mammalian cells [42] and transfection, are both carried out at equilibrium. These assays do not measure rates of import, but just the resultant of import and export localization at steady-state. This makes it difficult, if not impossible, to dissect the role of phosphorylation in cases where the post-translational modification does not completely stimulate or repress nuclear import. As often in biology, phosphorylation and other post-translational modification finely tune biochemical reactions by up- or downregulating basal levels of transport. Thus, a quantitative analysis of the role of phosphorylation on nuclear import is still in its infancy. In reviewing the literature on this topic, we have identified at least six ways by which phosphorylation can stimulate nuclear import. In contrast, there are only two characterized ways by which phosphorylation has been shown to down-regulate passage of cargos through the NPC. Both aspects of the phosphorylation-mediated regulation of nuclear transport will be discussed below.

## Up-Regulation of Nuclear Import by Phosphorylation

### Phosphorylation within an NLS enhances the binding affinity for the isoform importin α5

**Figure 3:**
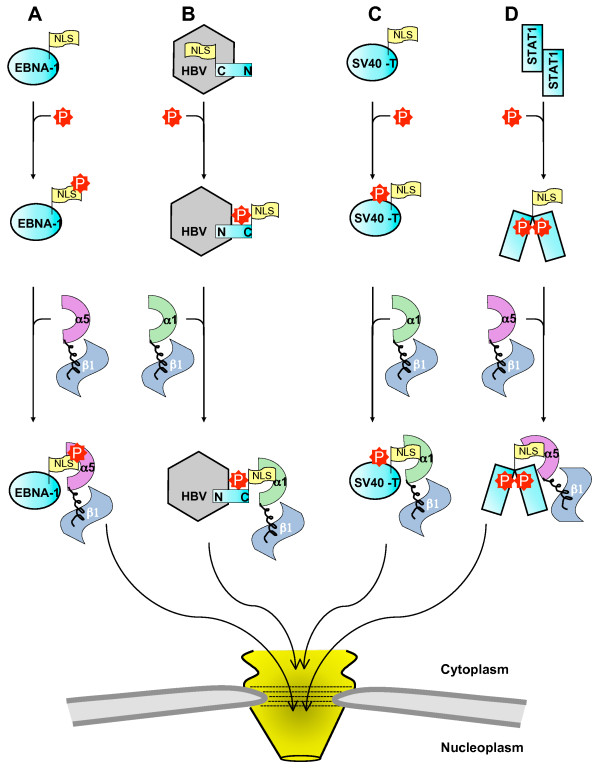
**Schematic representation of four distinct mechanisms by which phosphorylation can up-regulate importin **β**1-dependent nuclear import**. (**A**) Phosphorylation within the NLS of EBNA-1 enhances the binding affinity for importin α5. (**B**) Phosphorylation of the HBV core antigen causes a conformational change that positions the NLS on the exterior of the capsid, and hence promotes its nuclear import. (**C**) Phosphorylation of serines upstream of the NLS of SV40 large T-antigen enhances nuclear import likely by enhancing the cargo recognition by importin α1. (**D**) Phosphorylation of STAT1 at Tyr701 induces a conformational rearrangement that exposes a *non-classical *dsNLS that is bound specifically and with high affinity by importin α5. In all diagrams, import cargos and importin β1 are colored in cyan and blue, respectively while importin α1 is in green and α5 in violet. The NLS is depicted as a yellow flag and the phosphorylation site, or the kinase responsible for phosphorylation, is in red.

Perhaps the most intuitive way by which phosphorylation enhances nuclear import of a cargo is by increasing its affinity for a specific import factor. There are several well-documented examples of import cargos that fall under this category. One case is the Epstein-Barr virus (EBV) nuclear antigen 1 (EBNA-1) protein (Figure 3A), which is essential for the replication of EBV DNA in latently infected cells [43]. During infection EBNA-1 enters the nucleus of infected cells and functions as a transactivator of latent genome transcription [44]. It was shown that phosphorylation of Ser^385 ^in the EBNA-1 NLS (Figure 1B) up-regulates its nuclear transport by increasing the binding affinity for the import adaptor importin α5 (Figure 2C), which in turn recruits the receptor importin β1 [45].

Interestingly, replacement of Ser^385 ^with the phosphomimetic aspartic acid decreases the binding affinity for importin α5, demonstrating that the specificity of the response is dependent on the phosphate moiety. Additionally, biophysical studies using chemically synthesized peptides and isothermal titration calorimetry determined that phosphorylation of Ser^385 ^in EBNA-NLS increases the binding affinity for importin α5 by ~20-fold, compared to the unphosphorylated NLS (K_d _~3 μM versus 60 μM) [46]. Thus the phosphate moiety within EBNA-1 NLS functions as a direct binding determinant for importin α5; phosphorylation of Ser^385 ^within the NLS stimulates nuclear import by enhancing the binding affinity of EBNA-1 for the import adaptor importin α5.

### Phosphorylation enhances docking of cargos to the Nuclear Pore Complex

The human Hepatitis B virus (HBV) undergoes an elegant and tightly regulated mechanism of entry into the host nucleus to replicate its genome. A mature HBV particle contains an envelope with surface glycoproteins that surrounds an icosohedral capsid (composed of 180 or 240 copies of the 21 kDa core protein) containing the viral dsDNA genome, a viral polymerase and the host proteins Protein Kinase C (PKC) and hsp90 [47]. Progeny HBV particles assemble in the cytoplasm, but require the host RNA polymerase II in the nucleus for viral genome replication [48], making nuclear import of the genome a necessary step in viral replication. It was shown by Rabe *et al*. that the entire HBV core particle (32-36 nm diameter [49]) traverses the NPC in a phosphorylation-dependent manner [50]. The core protein displays an arginine-rich NLS at its C-terminus [51] (Figure 1B), which overlaps with a nucleic acid binding domain [52] and five serine phosphorylation sites [53]. The C-terminus of the core protein is buried inside the capsid [54] and interacts with the viral genome through the nucleic acid binding domain. The host PKC, also present in the infectious virion, phosphorylates the core protein at Ser^172^, thereby triggering a conformational change that exposes the core protein C-terminal NLS on the capsid surface (Figure 3B). Upon phosphorylation, the HBV core particle is recognized by an importin β1/importin α1 heterodimer and imported into the nucleus in a Ran and energy-independent manner [47]. Nuclear transport of the HBV core particle arrests at the nuclear basket [55], the capsid disassembles, and the viral genome is released into the nucleus and transcribed by the host RNA polymerase II. Core protein phosphorylation is clearly the key step in the nuclear import of HBV particles, but it is tightly coupled to the maturity of the viral genome. The host RNA polymerase II transcribes a pregenomic RNA, which is exported from the nucleus and trapped within newly forming capsids. Within the capsid, pregenomic RNA is then reverse-transcribed, and an incomplete complementary DNA strand is created. The genome maturity is not only dependent on the aforementioned events, but also on phosphorylation of the capsid proteins, which are thought to stabilize the HBV genome [56]. It was shown that the exposure of the core protein C-terminal NLS is directly proportional to genome maturity, and while immature virions are arrested at the NPC, only replication-competent capsids are imported into the nucleus [50].

### Phosphorylation upstream the classical SV40 NLS enhances its recognition by importin α1

The enhanced nuclear import of phosphorylated cargos does not always depend on direct interaction of the import machinery with the phosphate moiety. The first identified and likely one of the best-characterized NLSs is that of the large tumor antigen of simian-virus 40 (SV40) (Figure 1B). Upstream of the basic cluster, which spans residues 127-131, there are several phosphorylation sites (110-PS^111^S^112^DDEAAADS^120^QHAAPP**KKKRKV**G-133) (Figure 1B). It was reported that phosphorylation at S^111^/S^112 ^by protein kinase CK2 accelerates the nuclear import of SV40 large T-antigen 50-fold [57,58] and phosphorylation at an additional site, S^120^, by the double-stranded DNA-dependent protein kinase, further enhances nuclear import [59]. However, a crystal structure of importin α1 (lacking the IBB domain) in complex with a phospho-NLS peptide spanning region 109-133 of the SV40 large T-antigen showed that the phosphorylation sites do not make specific contacts with importin α1 (Figure 2B). Likewise, binding studies revealed that phosphorylation upstream of the SV40 T-antigen NLS does not increase the affinity of the NLS to importin α1 [60]. Interestingly, in the same crystal structure, it was observed that certain SV40 T-antigen residues upstream of the basic cluster also make specific contacts with importin α that are distinct from the binding sites of classical NLSs [61]. Based on these findings, it was proposed that phosphorylation upstream of the classical NLS might play a role in nuclear import by modulating the recognition of the NLS, rather than increasing the binding affinity of the NLS for importin α1 (Figure 3C). Furthermore, the observation that importin α1 does not bind the phosphate moiety directly further reinforces the idea that phosphorylation can play a regulatory role outside of the simple NLS-binding groove.

### Phosphorylation of STAT1 induces a conformational change that exposes a dimer-specific NLS

STATs (Signal Transducers and Activators of Transcription) form an important family of transcription factors that play a critical role in cellular viability, immune response, and development. There are seven known human STAT isoforms that share a fundamentally conserved multi-domain architecture composed of an N-terminal dimerization domain [62], a core consisting of a coiled-coil domain, DNA-binding domain, Src2 Homology domain (SH2) [63-65], and a C-terminal transactivation domain [66]. Each domain has critical roles in STAT activation, nuclear transport, or transcriptional activity. Consistent with their function of transcription factors, STATs have developed numerous ways to traverse the NPC and gain access to the nucleus; phosphorylation plays an essential role in this process.

The first STAT family member to be characterized, STAT1, mediates the innate immune response [67]. Upon stimulation of extracellular receptors, STAT1 is recruited to the cell surface and activated through tyrosine phosphorylation at position Tyr^701 ^[68]. Phosphorylation of STAT1 induces homodimerization through a reciprocal SH2-phosphoTyr interaction with another STAT1 monomer [68]. This dimerization leads to a structural rearrangement [69] of STAT1 that exposes a dimer-specific NLS (dsNLS) in its DNA-binding domain (Figure 1B) [70-73]. The STAT1 dsNLS differs from a classical NLS (cNLS) in numerous ways. First, the dsNLS is not recognized by importin α1, but as in the case of EBNA-1 phosphoNLS, by the isoform importin α5 (Figure 3D) [74]. Second, STAT1 does not bind only the major and minor NLS binding grooves of importin α5, but requires importin α5 C-terminal Armadillo repeats (ARM) 9 and 10 for high affinity binding [46]. The final and most unconventional characteristic of the dsNLS is that it only functions in the context of phosphorylated STAT1 [70]. Whereas classical NLSs can be removed from the context of the native protein and function *in trans *when attached to an exogenous protein, the STAT1 NLS is only functional *in cis*, within the context of the pSTAT1 tertiary structure. This was elegantly shown by Meyer *et al*. who studied a peptide spanning residues 376-427 of the STAT1 DNA-binding domain and found that it did not accumulate in the nucleus in a micro-injection nuclear import assay [70]. Because the STAT1 DNA binding domain is exposed in the active dimer of phosphorylated STAT1, the dsNLS can only act as a nuclear import signal within the context of phosphorylated STAT1. In addition, STAT1 can heterodimerize with STAT2 and STAT3 [67], and requires the dsNLS in the DNA-binding domain to accumulate in the nucleus. Interestingly, STAT2 cannot form homodimers or enter the nucleus on its own, suggesting that the dsNLS is a unique to STAT1 or STAT1-associated complexes [75].

### Phosphorylation unmasks an NLS through disruption of an NES

The extracellular signal regulated kinase (ERK) cascade has an important role in cellular development and phase transitions in the cell cycle [76]. The cytoplasmic bottleneck of this cascade is the ERK kinase, which accumulates in the nucleus 15 minutes after the mitogenic stimulus, and phosphorylates transcription factors critical for development [77]. ERK5 is an important member of the MAP kinase family that stimulates the transcriptional activity of c-Fos, c-Myc, MEF2 and numerous other factors involved in cardiovascular development and S-phase entry [76-79]. ERK5 (also termed Big MAP Kinase) is the largest of the ERK family members as it contains an extended C-terminal domain with two proline-rich regions, a transcriptional activation domain (TAD), and a bipartite NLS (Figure 1B), in addition to its N-terminal kinase domain [80]. Interestingly, ERK5 does not display a classical NES, but undergoes Crm1-mediated nuclear export [81,82]. Similar to the STAT1 dimer-specific NLS, ERK5 displays a conditional NES that only acts *in cis *[83]. In resting cells, the ERK5 N- and C-termini interact, which conceals the bipartite NLS, and creates an interface for Crm1 recognition [83]. The Map Kinase Kinase of ERK5 (MEK5) disrupts the ERK5 termini-dependent binding interface through phosphorylation of tyrosine and threonine residues in the Thr-Glu-Tyr (TEY) activation motif [84]. Upon phosphorylation, the putative NES is silenced, thereby exposing the bipartite NLS in the C-terminus, and hence promoting ERK5 nuclear accumulation [83]. It is unclear whether the ERK5 NES region recruits Crm1 directly, or via another NES-containing binding partner. Undoubtedly, phosphorylation plays a role in the nuclear import of ERK5 by altering the balance between NLS/NES signaling. In addition to multi-domain regulation of ERK5 nuclear transport, the N- and C-terminal domains are essential for transactivation of the transcription factor AP-1 [80]. AP-1 controls a number of cellular processes including proliferation, differentiation, and apoptosis. Upon activation, the ERK5 N-terminal kinase domain auto-phosphorylates the ERK5 C-terminal TAD, which has been shown to increase the transcriptional activity of AP-1 20-fold [80]. When expressed in the absence of the ERK5 kinase domain, the ERK5 TAD has no affect on AP-1 transcription. Notably, ERK5 is the only known cellular target of MEK5, and overexpression of MEK5 is associated with aggressive prostate cancer [85], cellular hypertrophy in cardiomyocytes [86], and impaired angiogenesis [87]. Thus, ERK5 phosphorylation plays a critical role in the coordination of inter- and intra-molecular binding interactions responsible for regulation of numerous cellular processes.

### Phosphorylation activates non-canonical transport signals that mediate nuclear import

**Figure 4:**
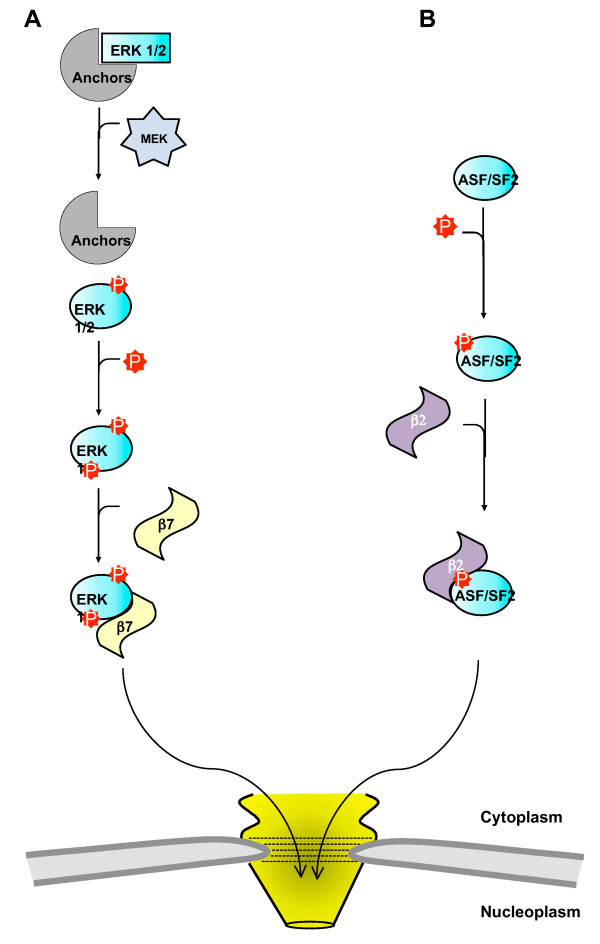
**Schematic representation of two phosphorylation-activated transport signals that confer nuclear import independent of importin β1**. (**A**) ERK1/2 (cyan) is sequestered in the cytoplasm by anchoring proteins (shown in grey). Upon phosphorylation at its TEY site, ERK1/2 undergoes a conformational change, causing it to dissociate from the anchoring proteins. This allows MEK access to the NTS which it phosphorylates. Upon NTS phosphorylation, ERK1/2 is recognized and imported into the nucleus by importin β7 (yellow). (**B**) The SR protein ASF/SF2 (cyan) is phosphorylated at Arg-Ser (RS) dipeptide repeats that function as an NLS for the import receptor importin β2 (also known as transportin), shown in purple.

In addition to basic NLSs (Figure 1B), several other small epitopes have been identified that when phosphorylated can promote nuclear import. For the purpose of this review, we will discuss two examples of these phosphorylation-activated transport signals: 1) the Nuclear Transport Signal (NTS) of ERK1/2 and 2) the arginine/serine (RS) dipeptide repeats of serine/arginine-rich proteins (SR proteins). The ERK family members ERK1 and ERK2 play a critical role in cellular development and cell cycle regulation. ERK1 and ERK2 are the cytoplasmic targets of the Ras-Ref-MEK-ERK signaling network, responsible for driving cellular proliferation through activation of transcription factors such as Fos and Elk-1 [88]. The MAPKK MEK1/2 is responsible for the activation of ERK1 and ERK2 [89]. Interestingly, MEK1/2 acts as a scaffold protein for ERK1/2, and anchors ERK in the cytoplasm under non-stimulating conditions [90,91]. Upon activation, MEK1/2 phosphorylates ERK1/2 at a TEY motif, which induces a conformational change in ERK1/2 and leads to its dissociation from MEK1/2 (Figure 4A). Until recently, the details of ERK1/2 nuclear transport were unclear. Unlike ERK5, which displays a bipartite NLS in its C-terminus [80], no obvious NLS or NES are discernable in the ERK1/2 primary sequence. Initially, it was believed that phosphorylated ERK1/2 entered the nucleus passively as a monomer, and that its more rapid, energy-dependent transport could involve dimerization and direct interaction with the NPC [92-94]. Recently, a novel NTS was identified for ERK1/2 in its kinase insert domain (Figure 1), which contains a characteristic Ser-Pro-Ser (SPS) motif. Upon stimulation, the SPS is phosphorylated and becomes functionally active. The phosphorylated motif is recognized by the nuclear transport receptor importin β7 [95] (Figure 4A), also a β-karyopherin implicated in the nuclear transport of two other proteins, MEK1 and SMAD3, which also contain SPS or TPT motifs [95,96]. Highlighting the importance of tightly regulated ERK1/2 phosphorylation in human biology, upregulation of MEK1/2 in brain tissue is linked to neurofibrillary degeneration in Alzheimer's disease [97].

Similar to NTS-containing proteins, serine/arginine-rich (SR) proteins rely on phosphorylation-dependent recognition of a small epitope for their nuclear import. SR proteins are fundamental to mRNA metabolism in the nucleus [98], and cycle between the cytoplasm and nucleoplasm depending on the level of transcription [99]. The prototypical SR protein ASF/SF2 is essential to genome stability, and depletion of this factor in metazoa was shown to induce G2 cycle arrest and apoptosis [100,101]. The RS domain of ASF/SF2 spans the C-terminal residues 198-248, and contains a series of Arg-Ser (RS) dipeptide repeats that can function as an NLS [99,102]. Interestingly, the RS-motif is only functional as an NLS when phosphorylated, as unphosphorylated ASF/SF2 localizes to the cytoplasm [103,104]. The importin β-like nuclear transport receptor importin β2 (also known as transportin) contains a unique internal domain responsible for the recognition of phosphorylated SR proteins [103,104] (Figure 4B). Like for importin β-1, nuclear import of phosphorylated SR proteins by importin β2 is expected to be regulated by RanGTP. With further investigation into the role of phosphorylation in nuclear transport, it is likely that various signaling clusters similar to the non-canonical NLSs of ERK1/2 and ASF/SF2 will be identified.

## Down-Regulation of Nuclear Import by Phosphorylation

### A phosphorylation-sensitive component of the nuclear transport machinery controls nuclear import

While phosphorylation of the nuclear envelope and NPC are necessary steps in cell cycle progression and the onset of mitosis, phosphorylation of the nuclear transport machinery in resting cells has a dramatic inhibitory effect on nuclear import. Kehlenbach *et al*. investigated the effect of phosphatase and kinase inhibitors on the nuclear transport of NLS-bearing cargos in a nuclear transport assay in digitonin-permeabilized cells [105]. This technique involves permeabilizing the cell membrane without disrupting the nuclear envelope so that the endogenous cytosol (soluble cellular proteins and transport factors) can be washed away and replaced with a controlled nuclear transport reaction mixture (e.g. purified importins, energy, etc.) [42] This allows dissecting the effect of individual components of the nuclear transport machinery on the nuclear transport of NLS- or NES-containing cargos. Kehlenbach *et al*. showed that the phosphatase inhibitors Okadoic Acid (OA) and microcystin dramatically inhibit nuclear import pathways mediated by transportin and importin β. Treatment with the broad-spectrum kinase inhibitor, staurosporine, was able to partially reverse the import inhibition, thereby suggesting that staurosporine-resistant kinases are also involved in the inhibition. They concluded that the phosphorylation targets were most likely nucleoporins in the NPC central channel because OA did not affect karyopherin recognition of NLS-cargo or the cellular localization of RanGTP. In addition, they suggested that the responsible kinases were both membrane associated and cytoplasmic. Interestingly, a recent investigation of the Picornaviruses EMCV and Theiler's murine encephalomyelitis virus showed the negative effects of the Leader protein (L-protein) on the nuclear transport machinery. The L-protein blocks nuclear transport in two known ways: 1) direct binding of Ran, which leads to misregulation of its activity [106], and 2) upregulation of cellular kinases, leading to the hyperphosphorylation of nucleoporins (specifically Nup62, Nup214, and Nup153) [107]. It will be interesting to define the exact kinase(s) responsible for Nup hyperphosphorylation, and whether the same kinase(s) is involved in the regulation of nuclear transport under normal physiological conditions.

### Cytoplasmic retention of cellular cargos by direct phosphorylation of an NLS

A simple way to retain cargos in the cytoplasm is by inactivating the NLS. This can be achieved either by direct binding and sequestering of the NLS, like the inhibitory protein of NF-kB (IKB) [108], or by phosphorylation of certain residues within the NLS. The latter is well documented for at least five cargos, two in humans and three in budding yeast, which will be discussed here.

The nuclear factor of activated T-cells (NFAT) is a well-characterized example of cargo retained in the cytoplasm by a phosphorylation in its NLS. First identified as a critical transcription factor for T-cell activity [109,110], NFAT is involved in numerous biological processes including the growth and development of muscle and neuronal cells as well as the immune response. There are four NFAT family members, which share highly conserved DNA-binding domains and serine- and proline-rich regions [110,111]. NFAT activity it tightly regulated by kinases and phosphatases in a calcium-dependent manner. Under normal physiological conditions, NFAT is localized to the cytoplasm due to phosphorylation of its serine-rich region (SRR2) [112], which overlaps with the NFAT NLS (Figure 1C). In addition to kinases that sequester NFAT in the cytoplasm, NFAT also contains a strong NES for Crm1-mediated nuclear export, which aids in its cytoplasmic localization [113]. Elevated cellular Ca^2+ ^levels stimulate Calcineurin, a Ca^2+ ^dependent serine phosphatase, which then binds to NFAT and dephosphorylates the NLS, making it accessible for recognition by the nuclear import machinery [114]. A drop in Ca^2+ ^levels or inactivation of Calcineurin by CsA or FK506, results in the phosphorylation of the NFAT NLS [115], which inhibits its nuclear accumulation. Calcineurin is a popular target of immunosuppressive drugs because its inactivation inhibits the ability of NFAT to enter the nucleus and stimulate an immune response. In addition to its role in the immune response, precise Calcineurin/NFAT signaling is required for axonal outgrowth of embryonic neurons [116], a process tightly linked to memory and learning [117].

The second important example of phosphorylation-mediated down-regulation of nuclear import that has important physiological consequences in humans is the parathyroid hormone related protein (PTHrP). This small secreted protein is best known for its role in chondrocyte maturation, but it is also expressed in a range of tumors as it has fundamental roles in cell cycle control and apoptosis [118,119]. The PTHrP gene yields three distinct alternative splice products, in a tissue specific manner that can display both paracrine and intracrine activities [120]. Its paracrine characteristics are attributed to the N-terminus of PTHrP, which is highly homologous to the secreted peptide hormone PTH, while PTHrP intracrine activities in the nucleus rely on its C-terminus [121]. After internalization by receptor-mediated endocytosis, PTHrP is rapidly transported into the nucleus, where it is involved in accelerating cell cycle and cell proliferation [122]. PTHrP displays a long non-classical NLS (ncNLS) between residues 66-97 (Figure 1C), which is recognized directly by importin β, independent of importin α [123]. Structural studies in complex with an N-terminal fragment of importin β revealed that the PTHrP-ncNLS binds HEAT repeats 2-11 of importin β HEAT, which are also responsible for binding to RanGTP and importin α [40]. The nuclear import of PTHrP is down regulated by the cell cycle-dependent kinases p33 and p34, which phosphorylate Thr85 in the ncNLS (Figure 1C) [124]. Interestingly, in the crystal structure of PTHrP-ncNLS bound to importin β, the site of phosphorylation at Thr^85 ^is located in close proximity to two well conserved tryptophan residues of importin β, which are known to be important for nuclear import of classical NLS cargos [125]. A phosphate moiety at this position could disrupt the interaction of the tryptophan side chains with the PTHrP-ncNLS, likely reducing the binding affinity for importin β and hence down-regulating nuclear import of PTHrP. However, since PTHrP is small enough to diffuse into the nucleus, phosphorylation in this case only slows down and does not completely obliterate the biological effect of PTHrP.

The transduction of extracellular signals to the nucleus displayed by higher eukaryotes occurs through analogous pathways in lower eukaryotes. Not surprisingly, phosphorylation plays a critical role in this process. The cellular localization of the transcription factor Msn2p in *Saccharomyces cerevisiae *is highly sensitive to stress response elements (STREs) [126]. Activation of Msn2p leads to the up-regulation of over 150 genes responsible for coping with environmental stresses such as osmotic shock, oxidative damage, chemical insult, and heat and nutrient depravation [127]. In particular, Msn2p nuclear transport under nutrient deprived conditions is regulated by the cAMP-dependent protein kinase (cAPK). The C-terminus of Msn2p contains four arginine- and lysine-rich clusters that function as a classical NLS [128]. These clusters overlap with serine phosphorylation sites (RRXS) targeted by cAPK (Figure 1C). Under normal physiological conditions, Msn2p is restricted to the cytoplasm due to phosphorylation of one or more of its basic clusters by cAPK, which inhibits importin α recognition and thus nuclear import of Msn2p [129]. Under glucose-deprived conditions, cAPK is down regulated, which leads to the accumulation of unphosphorylated Msn2p in the nucleus, and ultimately a transcriptional response to extracellular stress.

Similar to Msn2p, phosphorylation-mediated down regulation of nuclear import has been described for the S. cerevisiae transcription factor Pho4. Budding yeast has an extensive network of proteins involved in regulation of inorganic phosphate availability [130]. Upon phosphate starvation, dephosphorylated Pho4 is recruited to the nucleus where it in turn activates genes that respond to the levels of cellular phosphate [131]. When there is a supply of inorganic phosphate available to the cell, Pho4 is phosphorylated on five separate serine/proline motifs [132]. Two of these motifs are required for nuclear export, as binding to Pho4's export factor, Msn5, is strictly dependent on the phosphorylation state [131,133]. A third serine/proline phosphate site is contained within Pho4's NLS (Figure 1C). Upon phosphorylation, the import factor Pse1 is no longer able to bind to Pho4 [131,133]. Together, the increase in nuclear export signal potency and hindrance of nuclear import synergize to assure that Pho4 is retained in the cytoplasm during times of inorganic phosphate availability.

Another example of down-regulation of nuclear import by phosphorylation was reported for the S. cerevisiae transcription factor Swi6. Swi6p regulates the expression of several genes during the G1 phase of the cell cycle [134,135], when it enters the nucleus by active signal-mediated transport. Interestingly, hypophosphorylation of serine 160 of Swi6p located immediately upstream of the NLS correlates with Swi6p nuclear import [135] (Figure 1C). In contrast, hyperphosphorylation of serine 160 during the other phases of the cell cycle correlates with cytoplasmic localization [135]. Thus, the cell cycle-dependent phosphorylation of Swi6p correlates with its localization, and ultimately expression of downstream genes [135]. Using *in vitro *solution binding assays, Harreman et al. demonstrated that phosphorylation of serine 160 upstream of Swi6-NLS causes a 4-fold decreased binding affinity between the Swi6 and importin α1 [136]. A similar drop in affinity for observed by replacing serine 160 with a glutamic acid (S160E-NLS), which functions as a phosphomimetic. In addition, a GFP-tagged Swi6 S160E-NLS mutant showed a decreased rate of nuclear import when compared to wild type GFP-Swi6-NLS supporting the hypothesis that phosphorylation directly adjacent to an NLS decreases the binding affinity for importin α1, which correlates with reduced nuclear import efficiency of the phosphorylated cargo.

## Role of Phosphatases in Regulating Phosphorylation-Dependent Nuclear Transport of Stat1

### Cellular Phosphatases target STAT1

Because STAT1 is the most well studied example of phosphorylation-dependent nuclear transport, there has been much interest in the identification of phosphatases that counteract STAT1 activation. IFN signaling cascades undergo rapid activation-inactivation cycles that require tight regulation. The IFNγ signaling pathway is negatively regulated in at least three ways: 1) suppressors of Cytokine Signaling inhibition of JAKs and the IFNγ receptor at the cell membrane [137], 2) nuclear localization of the Protein Inhibitor of STAT1 factor [138], and 3) tyrosine and dual-specificity phosphatases that dephosphorylate STAT1 at Tyr^701^. One of the first phosphatases identified in the regulation of STAT signaling was the T-cell protein tyrosine phosphatase (TC-PTP). The TC-PTP gene is alternatively spliced, yielding a longer ER-associated form TC48, and a nuclear form TC45 [139,140]. The nuclear form of TC-PTP is responsible for the cooperative deactivation of STAT1 when it is released from target DNA [141]. Upon dephosphorylation by TC45, STAT1 undergoes Crm1-mediated nuclear export and participates in successive activation-inactivation cycles in stimulated cells [142]. In addition to its role in STAT1 deactivation, TC45 has also been implicated in the dephosphorylation of STAT3 in the nucleus.

The Src homology domain (SHP) family of dual-specificity phosphatases (SHP-1 and SHP-2) also plays a regulatory role in STAT nuclear localization; SHP's have a unique dephosphorylation mechanism, utilizing two tandem N-terminal SH2-domains (N-SH2 and C-SH2), a tyrosine phosphatase (PTP) domain and two C-terminal Tyr phosphorylation sites. Structural investigations of SHP-2 reveal an auto-inhibitory mechanism of the PTP domain by the N-SH2 in the absence of a pTyr substrate [143]. When the C-SH2 domain recognizes a bipartite pTyr ligand it binds one pTyr, and the N-SH2 domain binds the other pTyr, reversing the autoinhibition of the phosphatase. The SHP Tyr residues in its C-terminus can also represent the bipartite ligand when these residues are phosphorylated. While SHP-1 is primarily expressed in hematopoetic cells, SHP-2 is constitutively expressed in most cell types, and regulates a number of pathways including JAK/STAT, MAP Kinase, NFκB, and PI3 kinase [144]. SHP-2 negatively regulates the JaK/STAT pathway through dephosphorylation of a number of receptor tyrosine kinases at the cell membrane [67], Jak2 in the cytoplasm [145], and STAT1 in the nucleus [146]. Each of these substrates contains two or more proximal phospho-tyrosine residues most likely targeted by the SHP domain architecture. The examples described above only represent a few of the many ways that native phosphatases maintain balance within the Jak/STAT pathway, and control the nuclear transport of STAT1.

### Viral Phosphatases target cytokine signaling pathways

**Figure 5:**
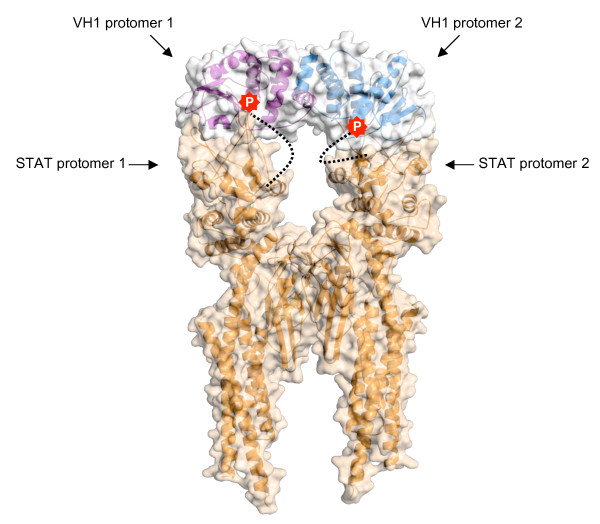
**Structural model of the *Vaccinia *virus phosphatase, VH1 (pdb **3CM3**) in the act of dephosphorylating activated STAT1 (pdb **1BF5**)**. Surface representation of the dimeric phosphatase VH1 modeled in the act of dephosphorylating phosphorylated STAT1 core (in gray and orange, respectively). Ribbon diagram of VH1 and phosphorylated STAT1 are overlaid to their surface. The two active sites within VH1 are spaced 39Å apart. STAT1 flexible moiety connecting phosphorylated Tyr^701 ^to the SH3 domains is modeled as a black dashed line; the two phosphorylated Tyr^701 ^(shown in red) are modeled inside each of VH1 active sites. It is intriguing to speculate that VH1 specificity for activated STAT1 may be mediated by a dimeric quaternary structure. Accordingly, VH1 positions two active sites in the correct three-dimensional complementarity to recognize and dephosphorylate activated STAT1.

Out of necessity, viruses have developed a number of mechanisms to evade the host innate immune response and neutralize cytokine signaling pathways, specifically the Jak/STAT pathway. Many virus families encode enzymes responsible for the evasion of host defense mechanisms, but few viruses like *Poxviridae *(e.g. *vaccinia*, *variola *virus etc) are known to encode phosphatases. Members of the *Poxviridae *family of double stranded DNA viruses carry a large, linear, genome of approximately 200-kbp, and assemble entirely in the cytoplasm of host cells [147]. *Poxviridae *encodes the dual-specificity phosphatase VH1, which is highly conserved among poxviruses and essential for the viability of *Vaccinia *virus in tissue cultures [148]. VH1 is expressed in late-stage of viral infection and an estimated 200 copies of VH1 are packaged within the virion [148]. VH1 has at least two important functions in the Vaccinia virus lifecycle: first, it dephosphorylates two-virion membrane-associated factors A17 [149] and A14 [150], *in vivo*. Second, VH1 was shown to deactivate host IFNγ signaling through dephosphorylation of specific tyrosine and serine residues required for STAT1 nuclear import and transcriptional activity [151]. It has been proposed that VH1 targets phosphorylated STAT1 (pSTAT1) in the cytoplasm as pSTAT1 bound to DNA is resistant to VH1 dephosphorylation, *in vitro *[152]. Moreover, STAT1's dedicated import adaptor importin α5 efficiently competes with VH1 for STAT1 dephosphorylation, suggesting both importin α5 and VH1 encounter pSTAT1 in the cytoplasm prior to its nuclear import [152]. Dephosphorylation of STAT1 at Try701 blocks carrier mediated nuclear accumulation of STAT1 [19] but does not affect the carrier independent import of unphosphorylated STAT1, which occurs via direct binding to nucleoporins [153]. Like phosphorylated STAT1 [69], VH1 displays a dimeric quaternary structure [152]. Interestingly, the active sites of the VH1 monomers are spaced 39Å apart, which is equal to the distance between the phospho-Tyr residues seen in the crystal structure of pSTAT1 bound to DNA [63]. This raises the intriguing possibility that VH1 dimeric quaternary structure has been specifically evolved as a dedicated solution to optimize recognition and dephosphorylation of STAT1 (Figure 5). This, in turn, prevents nuclear import of STAT1 and transcription of interferon-γ genes, thereby blocking the antiviral response.

In the past decade, an increasing number of phosphatases have been implicated in the deactivation of JaK/STAT signaling through the dephosphorylation of STAT1 or its upstream effectors. For example, two Flaviviruses, the mosquito-borne flavivirus JEV and the tick-borne TBE both encode the protein NS5, which was shown to disrupt IFN signaling. While NS5 does not display a structurally conserved phosphatase motif, treatment of infected cells with the PTP inhibitor sodium orthovanadate restored IFNγ and IFNα signaling [154,155]. This suggests that although NS5 is not a phosphatase itself, it may function by upregulating the activity of endogenous phosphatases that dephosphorylate members of the JaK/STAT pathway. A recent investigation of the Dengue virus NS5 protein and STAT2 showed that NS5 directly binds STAT2, inhibiting STAT2 phosphorylation and IFNα signaling (mediated by STAT1/2 heterodimers) [156], but this effect did not affect IFNγ signaling (mediated by STAT1 homodimers). Although it remains unclear how Flavivirus-specific NS5 proteins inhibit IFN signaling pathways, one shared characteristic seen in different viruses is that evasion of the immune response occurs by blocking STAT nuclear import, and hence, STAT-mediated antiviral response.

## Conclusions

It has been 55 years since the groundbreaking study on the conversion of phosphorylase b to phosphorylase a, by Fisher and Krebs, that first emphasized the importance of reversible phosphorylation in biology [157]. Today, we know that phosphorylation is the key regulatory step in signaling pathways responsible for cellular development and differentiation, cell cycle control, metabolism, and the immune response. It acts not only as a veritable on and off switch for protein activity, but also as a precise mechanism of redirecting a protein's cellular localization, and thus its function in the cell. As described in this review, the role of phosphorylation in the nuclear transport of cargos has only now begun to be elucidated. We have discussed key examples of the different mechanisms by which this occurs. In this final section, we will try to summarize the broad significance of this novel regulatory mechanism. Although the phosphorylated cargos described in this review are seemingly diverse, some general rules emerge comparing their function. Nearly all known cargos whose nuclear import is regulated by phosphorylation act directly (or indirectly) on the control of gene expression. Certain cargos are transcription factors (e.g. STAT1, NFAT, Msn2p, Pho4, Swi6) that directly bind DNA and trigger expression of critical genes, or kinases that specifically phosphorylate transcription factors critical for development (e.g. ERK proteins). Other cargos (mainly viral) function as transactivators of genome transcription (EBNA-1), or like SV40 T-large antigen, bind to important cellular proteins such as p53 and retinoblastoma protein (pRb), and thereby are capable of transforming a variety of cell types. Finally, other cargos such as SR proteins or PTHrP [158] are linked to RNA metabolism. Thus, phosphorylation of these import cargos alters their nuclear availability and thereby functions as a liaison between extracellular stimuli and gene expression in the nucleus. This provides a novel and useful level of regulation that allows turning genes on and off when cellular and developmental conditions demand it. By controlling the availability of transcription factors in the nucleus, cells respond to extracellular stimuli and trigger proliferation or defense mechanisms. Intuitively, the regulation of nuclear import and nuclear availability of transcription factors and certain proteins that directly affect gene expression is vital for cell proliferation and linked to human disease. For instance, STAT1 is becoming increasingly important in cancer biology [159-163]. Although the IFN-γ/STAT1 signaling is usually connected with anti-viral response and pro-apoptotic tumor-suppressor functions, constitutively activated IFN-γ/STAT1 pathway has been recently associated with aggressive tumor phenotypes [163]. For instance, high level of nuclear phosphorylated STAT1 is hallmark of resistance to IFN-γ and radiation therapy, which is commonly acquired during radiotherapy treatment and accounts for many treatment failures [160]. In this respect, nuclear entry of activate STAT1 is also of potential pharmacological interest. Small molecule inhibitors that reduce accumulation of phosphorylated STAT1 in the nucleus would function as useful anticancer agents and likely prevent radiation resistance. Similarly, PTHrP's role as a growth/malignancy factor clearly correlates to its ability to localize in the nucleus/nucleolus and thereby delay apoptosis [158]. In conclusion, there is little doubt that phosphorylation adds another level of regulation to the already tightly control trafficking of proteins between the nucleus and cytoplasm. The challenge is now to expand the analysis of import cargos that are phosphorylated and develop more quantitative methods to measure how phosphorylation affects the kinetic of nuclear localization as well as protein turn-over, *in vivo*. This is likely to have a very significant impact on our ability to understand how extracellular stimuli (e.g. cytokines, hormones, environmental stress) affect, modulate and control gene expression in the cell nucleus.

## List of Abbreviations used

NLS: nuclear localization signal; NPC: nuclear pore complex; NE: nuclear envelope; ONM: outer nuclear membrane; INM: inner nuclear membrane; IBB: importin β binding; NES: nuclear export signal; NUP: nucleoporin; EBV: Epstein-Barr virus; EBNA-1: Epstein-Barr virus nuclear antigen 1; HBV: Hepatitis B virus; PKC: protein kinase C; STATs: signal transducers and activators of transcription; SH2: Src2 Homology domain; dsNLS: dimer-specific NLS; cNLS: classical NLS; ncNLS: non classical NLS; ARM: armadillo; ERF: extracellular signal regulated kinase; TAD: transcriptional activation domain; TEY: Thr-Glu-Tyr; NTS: Nuclear Transport Signal; SR protein: serine/arginine-rich proteins; SPS: Ser-Pro-Ser; OA: okadoic acid; IKB: inhibitory protein of NF-kB; NFAT: nuclear factor of activated T-cells; SRR2: serine-rich region; PTHrP: parathyroid hormone related protein; STREs: stress response elements; cAPK: cAMP-dependent protein kinase; TC-PTP: T-cell protein tyrosine phosphatase; PTP: protein tyrosine phosphatase; SHP: SH2-containing tyrosine phosphatase; pSTAT1: phosphorylated STAT1; IFN: interferon; JaK: Janus Kinase; PIAS: Protein Inhibitor of STAT1; SOCS: Suppressor of Cytokine Signaling; VH1: *Vaccinia *virus H1 gene product; JEV: Japanese Encephalitis Virus; TBE: Tick-Borne Encephalitis Virus.

## Competing interests

The authors declare that they have no competing interests

## Authors' contributions

JN wrote the initial draft of the manuscript; KL expanded and revised the manuscript, and generated all figures. GC revised both text and figures critically for important intellectual content; all three authors have given final approval of the version to be published.
